# Calibration of Self-Reported Confidence and Accuracy of Large Language Models in Medical Question Answering

**DOI:** 10.1007/s10916-026-02430-0

**Published:** 2026-06-26

**Authors:** Sebastian Daniel Boie, Florian Reis, Nicolas Frey, Elias Grünewald, Felix Balzer

**Affiliations:** 1 Institute of Medical Informatics, Charité – University Medical Center Berlin, corporate member of Freie Universität Berlin and Humboldt-Universität zu Berlin, Charitéplatz 1, 10117 Berlin, Germany; 2 Department of Anesthesiology and Intensive Care Medicine (CCM/CVK), Charité – University Medical Center Berlin, corporate member of Freie Universität Berlin and Humboldt-Universität zu Berlin, Berlin, Germany

**Keywords:** Large language model, Calibration, Confidence, Accuracy

## Abstract

Objective: To evaluate the alignment between self-reported confidence of large language models (LLM) and their accuracy in answering medical multiple-choice questions. Materials and Methods: We prompted six LLMs (GPT-5, GPT-5-mini, GPT-5-nano, GPT-4o, Claude Sonnet 4.5, and Gemini 2.5) to answer MedMCQA items and report confidence scores. Based on 12,000 LLM responses, we calculated Expected Calibration Errors (ECE) by averaging absolute differences between observed accuracy and predicted confidence. Results: Mean ECE differed by model (Claude Sonnet 4.5 best: 0.06; Gpt-4o worst: 0.127) and varied across specialties (“Skin” best: 0.041; “Social & Preventive Medicine” worst: 0.141). Accuracy of examined LLMs showed analogous variation between specialties. Discussion: Our results demonstrate that high accuracy does not guarantee reliable uncertainty estimation. We identified substantial heterogeneity across medical specialties, where pooled metrics masked a threefold ECE increase between best- and worst-performing domains. Conclusion: We recommend incorporating calibration reporting into LLM evaluations, as larger models exhibit improved “self-knowledge”, but uneven overconfidence persists.

**Supplementary Information:**

The online version contains supplementary material available at 10.1007/s10916-026-02430-0.

## Introduction

*“It ain’t what you don’t know that gets you into trouble; it’s what you know for sure that just ain’t so”* (Mark Twain). Large language models (LLMs) are increasingly used to answer medical questions and to assist in clinical reasoning, both in educational contexts and in decision-support applications [1]. Although their accuracy continues to improve, trustworthiness depends not only on accuracy but also on whether stated confidence accurately reflects the true likelihood of being correct, a property known as calibration [2].

Poor calibration is particularly hazardous in medicine: overconfidence can lead to unsafe recommendations, while underconfidence may undermine clinical utility and user trust [3]. Calibration has therefore become a central concept in assessing the reliability of AI systems. For generative systems, assessing calibration requires eliciting uncertainty in interpretable ways. Reliable confidence estimates are even more critical for LLMs because “explainable AI” techniques remain challenging to apply to LLMs, whose internal reasoning processes are largely opaque [4], particularly considering regulatory expectations for trustworthy AI under frameworks such as the EU AI Act, which mandates human oversight for high-risk AI systems [5].

Recent studies show that LLMs may “know what they know” when confidence is explicitly elicited, such as through direct self-reported confidence scores, but the models may systematically overestimate their correctness [6]. Evaluations likewise reveal poor out-of-the-box calibration, with potential improvement after explicit calibration or fine-tuning [7]. Alternative approaches such as token-probability–based metrics [8], sample-consistency proxies [9], and meaning-aware scoring methods [10] can provide valuable uncertainty estimates but are less interpretable to end-users.

Self-improving and instruction-tuned LLMs may experience calibration drift as alignment alters internal likelihood distributions. This highlights the importance of regular re-evaluation and transparent reporting of calibration metrics. Most prior work reports pooled calibration across domains, obscuring potentially large specialty-specific differences in how well LLM confidence reflects reality [11]. Given that medical specialties vary in language, reasoning demands, and training data representation, calibration may differ substantially by domain.

This study evaluates self-reported confidence of several state-of-the-art LLMs across medical specialties using the MedMCQA benchmark [12]. By quantifying Expected Calibration Error (ECE) and accuracy across 20 medical disciplines, we characterize both model- and domain-level variation in calibration. This specialty-resolved analysis extends existing research on LLM uncertainty in medicine and provides actionable insights for selecting, benchmarking, and deploying LLMs.

## Methods

### Dataset and task

We used the publicly available MedMCQA benchmark (undergraduate/MBBS-style multiple-choice questions (MCQs)) with a 20-specialty taxonomy. This large-scale benchmark dataset contains over 194,000 MCQs. We randomly sampled 100 questions per specialty; difficulty of the questions was not assessed. 

We evaluated accuracy by comparing model answers with ground truth, and elicited confidence for six LLMs on this sample. This amounts to 2000 questions per model and 12,000 model responses overall.

### **Models and prompting**

We evaluated six LLMs from three different vendors: GPT-5, GPT-5-mini, GPT-5-nano, GPT-4o (all OpenAI), Gemini 2.5 (Google), and Claude Sonnet 4.5 (Anthropic). For each sample, the LLMs produced an answer as well as a self-reported confidence from 0 to 100, using a fixed instruction set with a one-line confidence elicitation. For the OpenAI models, we used the API (version 1.106.1) with default values (temperature = 1, top_p = 1, presence_penalty = 0.0, frequency_penalty = 0.0). For Gemini 2.5, we used the Google Gen AI API (version 1.41.0) with default values, and for Claude Sonnet 4.5 we used the Anthropic API (version 0.69.0) with default values (temperature = 1, top_p = 0.99). We followed a two-step established prompt [13]:

- Original prompt: “{question} Options: {…}. You are an experienced physician. Explain your answer and provide the correct option in the following format: Answer: [Letter]”.
- Confidence elicitation prompt: “{Original prompt} {answer}. You are an experienced physician. Your task is to rate the uncertainty of the proposed answer on a score from 0 to 100, where 0 represents definitely uncertain and 100 represents definitely certain. Please, only answer with your score.”

### **Primary metric: Expected Calibration Error (ECE)**

evaluates how well confidence values align with true outcomes by binning n items into M bins (B) by confidence and averaging the absolute differences between the bin’s mean confidence (conf) and empirical accuracy (acc):

$$\:ECE=\:{\sum\:}_{m=1}^{M}\frac{{B}_{m}}{n}|acc\left({B}_{m}\right)-conf({B}_{m})|$$

Intuitively, ECE quantifies the average mismatch between a model’s stated confidence and its actual number of correct answers per binned confidence level. Lower ECE indicates better calibration. We mapped self-reported confidence to probability (score/100) and computed ECE per model and per specialty. A model is defined as overconfident in a given bin if the empirical accuracy falls below the confidence midpoint. Consequently, a model is considered underconfident if its empirical accuracy is above the confidence midpoint (cf. Supplementary Fig. 1).

### **Additional outcome: accuracy**

We summarized accuracy (%) by specialty×model to complement calibration findings.

### **Analysis approach**

We report descriptive comparisons (means, rank ordering, figures). Given the high number of specialties/conditions and our exploratory scope, we report effect sizes and visualizations without p-values. To characterize the range of specialty-level calibration, we additionally report mean ECE for the best- and worst-performing specialty (lowest and highest mean ECE across models), alongside their ratio as a descriptive summary

## Results

**Fig. 1:**
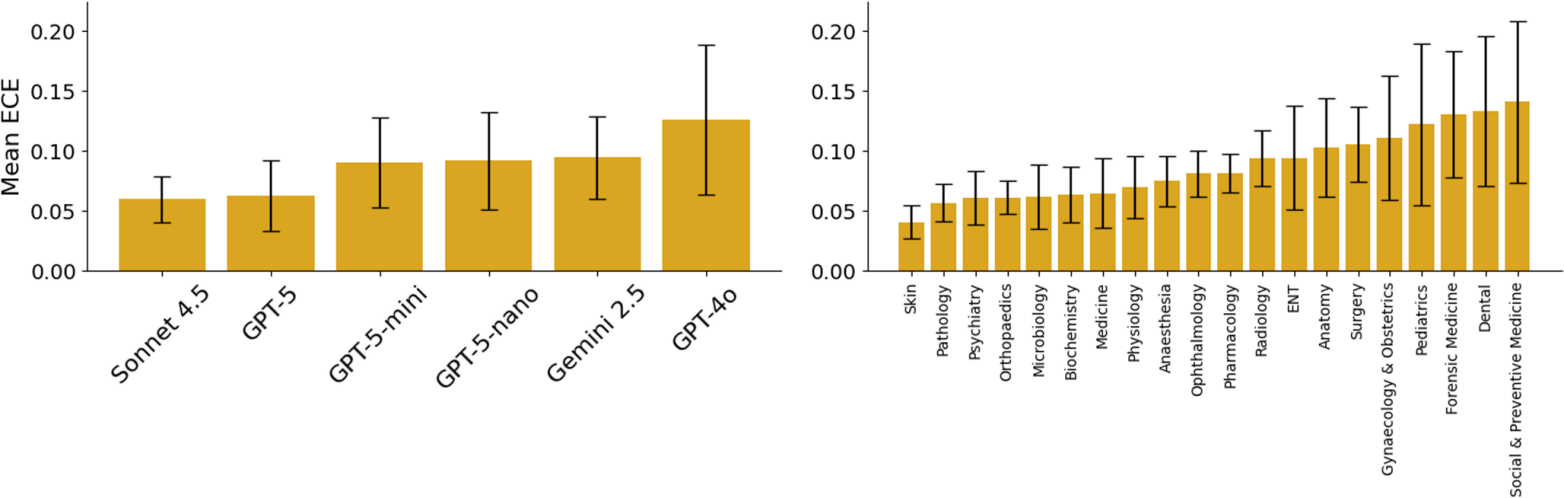
Mean expected calibration error by model (left panel) and by specialty (right panel). The heights of the bars represent mean values, and the whiskers represent the standard deviation. Lower ECE is better.

Accuracy and calibration varied substantially across models and specialties. Models performed best on “Skin”, which showed the highest accuracy and lowest ECE, whereas “Social and Preventive Medicine” showed the lowest accuracy and poorest calibration (cf. Fig. 1). Across all models, mean ECE by specialty ranged from 0.041 to 0.141, representing an approximately threefold difference between the best- and worst-calibrated domain.

Calibration curves for the specialties “Skin” and “Social and Preventive Medicine” are shown in Supplementary Fig. 1. In “Social and Preventive Medicine”, models were systematically overconfident, as indicated by the calibration points that fell below the diagonal line of perfect calibration.

GPT-5 showed strong accuracy and good calibration, whereas GPT-5-mini and GPT-5-nano exhibited lower accuracy and poorer ECEs. Gemini 2.5 achieved the second-highest accuracy but was overconfident (as defined in the methods section), resulting in comparatively poor calibration. Confidence scores per model stratified by correctness is shown in Supplementary Fig. 2.

Despite ranking third in accuracy, Claude Sonnet 4.5 demonstrated the best calibration overall (cf. Table 1).

**Table 1:** This table shows mean accuracy (higher is better) and mean ECE (lower is better) per model averaged across specialties and the standard deviation

Model	Accuracy (SD)	Rank accuracy	ECE	Rank ECE	
Claude Sonnet 4.5	88.6 (4.7)	3	0.06 (0.019)	1	
GPT-5	91.1 (4.3)	1	0.063 (0.030)	2	
GPT-5-mini	88.6 (4.7)	4	0.091 (0.038)	3	
GPT-5-nano	84.6 (4.9)	6	0.092 (0.040)	4	
Gemini 2.5	90.4 (3.3)	2	0.094 (0.034)	5	
GPT-4o	85.7 (6.5)	5	0.127 (0.063)	6	

A comparison of models by subtracting ECEs revealed that GPT-5 and Claude Sonnet 4.5 were better calibrated than the other models. Gemini 2.5 calibration was not superior to the smaller GPT-5-mini and -nano models, and GPT-4o’s calibration was inferior overall (cf. Supplementary Table 1).

Comparisons consistently showed that GPT-5 and Claude Sonnet 4.5 were the most reliably calibrated models across specialties, while GPT-4o and Gemini 2.5 exhibited the largest calibration deficits.

## Discussion

Our study indicates that current LLMs exhibit imperfect calibration in medical question answering, both across model architectures and medical specialties. While accuracy is frequently used as the primary proxy for utility, we show that high accuracy does not guarantee reliable uncertainty estimation. For instance, while Gemini 2.5 achieved the second-highest accuracy, it ranked poorly (5th) in calibration due to overconfidence. In contrast, Claude Sonnet 4.5 demonstrated the best alignment between confidence and correctness despite ranking third in accuracy. This confirms prior observations that LLMs tend toward overconfidence [7, 13] but adds a critical nuance: calibration is not uniform. We observed that scaling within a model family (OpenAI’s GPT-5 series) improved calibration, suggesting that larger parameter counts correlate with better “self-assessment”. It should be noted that self-reported confidence is not a standardized internal measure: each model expresses certainty differently, and direct comparisons across model families may therefore reflect differences in confidence expression as much as differences in true calibration. We interpret cross-model comparisons at the behavioral level (i.e., whether externally communicated confidence aligns with empirical accuracy under a standardized prompt).

A key aspect is the distinction between “being right” (accuracy) and “knowing you are right” (calibration). GPT-4o, widely used in clinical experiments, showed the poorest calibration (ECE 0.127) among models tested. Conversely, the superior calibration of Claude Sonnet 4.5 suggests that some training methodologies may better preserve internal uncertainty representations. An intra-vendor comparison of the GPT-5 series indicates that smaller model sizes may degrade calibration performance more quickly than accuracy. This is a factor that should be considered when deploying lightweight models.

For LLM-assisted clinical reasoning, we hypothesize that a model which accurately flags its own uncertainty may be preferable to a more accurate model that produces overconfident erroneous outputs. This hypothesis is supported indirectly by evidence that explicitly communicated uncertainty reduces user overreliance on LLM-generated answers [15]. We note, that this applies specifically to generative LLMs used in advisory roles; classical decision support systems typically rely on predictive rather than generative AI, and calibration requirements in those contexts may differ substantially.

This study offers evidence that LLM calibration is subject to specialty. We found a threefold difference in calibration error between the specialty with the best performance (ECE 0.041) and the worst performance (ECE 0.141). While visual and pattern-recognition heavy fields like Dermatology and Pathology showed high accuracy and tight calibration, possibly due to overrepresentation in training data and precise ground truth labelling, fields requiring complex contextual interpretation or those with potentially more ambiguous ground truths, such as “Social & Preventive Medicine”, suffered from overconfidence. However, these results may be influenced by other factors, such as question difficulty or question style (fact recall vs. opinion-based).

One further explanation for the observed model-level differences may be that vendor-specific training and alignment influence how models translate uncertainty into numeric confidence. Accordingly, differences in ECE may partly reflect model-specific use of the 0–100 scale, with some models expressing certainty more conservatively, regardless of their actual performance. Note that these explanations are given post-hoc and not tested, as question difficulty and question style were not controlled in the analysis.

Our findings also imply that “pooled” or global calibration metrics can mask potential safety risks [14]. Based on our results, a clinician relying on an LLM for medical advice on a topic related to “Preventive Medicine” may face a higher risk of “confident errors” than for dermatology-related questions. Although our analysis is descriptive, the magnitude of the observed calibration differences is considerable. Inter-model differences reached up to 0.064 (GPT-5 vs. GPT-4o), and inter-specialty differences reached 0.10.

An ECE of 0.10 indicates a systematic gap of 10% points between stated confidence and actual correctness, which translates into a substantial rate of “confidently wrong” recommendations, at a rate of at least one in ten decisions.

Current evaluations often treat medical knowledge as a uniform entity. Our results suggest adopting a broader perspective by including domain-specific calibration reporting. Rather than providing aggregate scores, ECE (or similar metrics) should be reported per clinical specialty. If well-calibrated, such scores could trigger a “human-in-the-loop” review or prompt the model to abstain from answering, when model confidence drops below a safety limit. It should be acknowledged, that confidence-based abstention involves an essential trade-off: lowering the confidence threshold reduces the rate of confidently wrong answers but simultaneously increases the rate of withheld correct answers. The optimal threshold is therefore not universal.

To our knowledge, this is the first study to investigate the interplay between accuracy and self-reported confidence of current LLMs stratified by medical specialties. Self-reported confidence has the advantage of being directly interpretable to end-users and requires no access to token probabilities or model internals, in contrast to token-probability–based calibration or consistency-based proxies (9). Recent work shows that communicating uncertainty to end users can reduce overreliance on LLM-generated answers [15], underscoring the importance of making model confidence visible and communicating it in an interpretable manner.

## Limitations

Our reliance on MedMCQA may not capture performance in open-ended clinical reasoning or real-world diagnostic scenarios. Furthermore, the non-deterministic nature of LLMs means that their calibration may drift. We only tested proprietary LLMs, whereas open-weights models may demonstrate comparatively inferior calibration. Our descriptive analysis should be complemented by conducting formal hypothesis testing in future experiments.

## Conclusion

The calibration of accuracy and self-reported confidence varies among LLMs and medical specialties. While larger models generally have better “self-knowledge”, high accuracy can coexist with overconfidence. Therefore, awareness of domain- and model-specific calibration profiles is useful for a balanced interpretation of LLM outputs in medical contexts. We recommend that future benchmarks prioritize calibration results alongside accuracy and other established metrics.

## Supplementary Material

Below is the link to the electronic supplementary material.

Supplementary Material 1

## Data Availability

All data to replicate the results of this manuscript will be uploaded to ZENODO upon acceptance.
