# Supplementary material for: Calibration of Self-Reported Confidence and Accuracy of Large Language Models in Medical Question Answering
Source: J Med Syst. 2026 Jun 26;50(1):103. doi: 10.1007/s10916-026-02430-0 (PMC13309378; doi:10.1007/s10916-026-02430-0)
Supplement: Supplementary file 1 [file 10916_2026_2430_MOESM1_ESM.docx]

# Appendix

Supplementary Figure 1 Calibration plots for the specialties of “Skin” (left) and “Social & Preventive Medicine” (right). The ordinate shows the empirical accuracy, and the abscissa shows the mean predicted confidence (top-1), which is the mean confidence value of predicted model answers. The dashed lines in our visualizations represent perfect calibration. Values above the line indicate model underconfidence, while values below the line indicate model overconfidence. The inset shows elicited confidence values across models. 

A screenshot of a graph  AI-generated content may be incorrect.

Supplementary Figure 2 Confidence by model stratified on answer correctness.

Supplementary Table 1 Comparison of ECE in pairwise model comparisons. Signed differences are shown and 95% confidence intervals obtained by bootstrapping with 5000 samples.

| Model 1 | Model 2 | Difference ECE (95% CI) | Better Calibrated model |  |
| --- | --- | --- | --- | --- |
| Gemini 2.5 | GPT-4o | -0.032 (-0.055, -0.012) | Gemini 2.5 |  |
| Gemini 2.5 | GPT-5 | 0.032 (0.016, 0.046) | GPT-5 |  |
| Gemini 2.5 | GPT-5-mini | 0.004 (-0.011, 0.018) | No significant difference |  |
| Gemini 2.5 | GPT-5-nano | 0.003 (-0.015, 0.02) | No significant difference |  |
| Gemini 2.5 | Claude Sonnet 4.5 | 0.035 (0.02, 0.05) | Claude Sonnet 4.5 |  |
| GPT-4o | GPT-5 | 0.064 (0.045, 0.083) | GPT-5 |  |
| GPT-4o | GPT-5-mini | 0.036 (0.019, 0.055) | GPT-5-mini |  |
| GPT-4o | GPT-5-nano | 0.034 (0.014, 0.055) | GPT-5-nano |  |
| GPT-4o | Claude Sonnet 4.5 | 0.066 (0.041, 0.095) | Claude Sonnet 4.5 |  |
| GPT-5 | GPT-5-mini | -0.028 (-0.042, -0.014) | GPT-5 |  |
| GPT-5 | GPT-5-nano | -0.029 (-0.047, -0.013) | GPT-5 |  |
| GPT-5 | Claude Sonnet 4.5 | 0.003 (-0.013, 0.019) | No significant difference |  |
| GPT-5-mini | GPT-5-nano | -0.001 (-0.015, 0.011) | No significant difference |  |
| GPT-5-mini | Claude Sonnet 4.5 | 0.031 (0.014, 0.048) | Claude Sonnet 4.5 |  |
| GPT-5-nano | Claude Sonnet 4.5 | 0.032 (0.016, 0.048) | Claude Sonnet 4.5 |  |
